# “The Terminology Might Be Ahead of Practice”: Embedding Shared Decision Making in Practice—Barriers and Facilitators to Implementation of SDM in the Context of Maternity Care

**DOI:** 10.1177/23814683231199943

**Published:** 2023-09-22

**Authors:** Alex Waddell, Denise Goodwin, Gerri Spassova, Peter Bragge

**Affiliations:** Monash Sustainable Development Institute, Monash University, Clayton, VIC, Australia; Safer Care Victoria, Victorian Department of Health, Melbourne, VIC, Australia; Behaviour Works Australia Health Programs, Monash Sustainable Development Institute, Monash University, Clayton, VIC, Australia; Department of Marketing, Monash Business School, Caulfield East, VIC, Australia; Monash Sustainable Evidence Review Service, Monash Sustainable Development Institute, Monash University, Clayton, VIC, Australia

**Keywords:** health policy research, hospital accreditation, health services research, implementation science, maternity care, shared decision-making

## Abstract

**Background.** It is a patient’s right to be included in decisions about their health care. Implementing shared decision making (SDM) is important to enable active communication between clinicians and patients. Although health policy makers are increasingly mandating SDM implementation, SDM adoption has been slow. This study explored stakeholders’ organizational- and system-level barriers and facilitators to implementing policy mandated SDM in maternity care in Victoria, Australia. **Method.** Twenty-four semi-structured interviews were conducted with participants including clinicians, health service administrators and decision makers, and government policy makers. Data were mapped to the Theoretical Domains Framework to identify barriers and facilitators to SDM implementation. **Results.** Factors identified as facilitating SDM implementation included using a whole-of-system approach, providing additional implementation resources, correct documentation facilitated by electronic medical records, and including patient outcomes in measurement. Barriers included health service lack of capacity, unclear policy definitions of SDM, and policy makers’ lack of resources to track implementation. **Conclusion.** This is the first study to our knowledge to explore barriers and facilitators to SDM implementation from the perspective of multiple actors following policy mandating SDM in tertiary health services in Australia. The primary finding was that there are concerns that SDM implementation policy is outpacing practice. Nonclinical staff play a crucial role translating policy to practice. Addressing organizational- and system-level barriers and facilitators to SDM implementation should be a key concern of health policy makers, health services, and staff.

**Highlights:**

- New government policies require shared decision making (SDM) implementation in hospitals.
- There is limited evidence for how to implement SDM in hospital settings.
- There are concerns SDM implementation policy is outpacing practice.
- Understanding and capacity for SDM varies considerably among stakeholders.
- Whole of system approaches and electronic medical records are seen to facilitate SDM.

## Graphical abstract

**Graphical Abstract:**
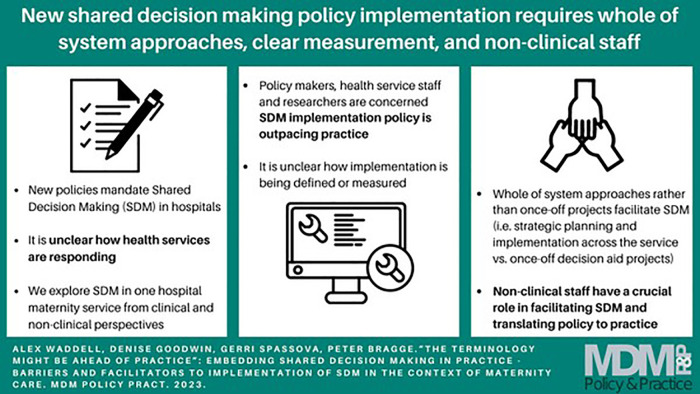
This is a visual representation of the abstract.

## Introduction

Shared decision making (SDM) involves an active two-way conversation between clinician and patient (and/or their carer or family member).^1,2^ The clinician provides information about treatment options, risks, and benefits, while the patient provides information about their own values, preferences, and goals.^1–4^ SDM implementation has been shown to reduce care variation, increase communication between patients and clinicians,^5^ and in some cases increase quality of life following treatment.^6^ Patients who experience higher levels of SDM participation have better reported health outcomes^7^ and decreased health care utilization compared with patients who have a lower level of participation in SDM.^5,7^

Despite the promising evidence, SDM integration into routine practice has been slow and piecemeal.^8^ Health policy is one of many mechanisms used to encourage or mandate implementation.^8,9^ SDM is now an important part of health policy objectives and instruments in several different countries including the United States, Canada, the United Kingdom, and Australia^10–13^ (among others).

Policies promoting SDM vary between jurisdictions in their form and application. SDM in the United Kingdom is supported by an integrated national policy that sets clear recommendations for SDM implementation supported by a centralized department.^14^ In the United States and Canada, SDM policies are piecemeal and decentralized with policy development and implementation left to the discretion of states, provinces, and territories.^10,12^ In Canada, SDM is promoted through education and training, development of resources such as patient decision aids, and funding streams promoting SDM research.^10^ Similar policy levers are used in the United States; however, given their move toward value-based health care (i.e., health care providers are paid based on patient outcomes^14^), insurers incentivize clinicians to practice SDM for specific treatment options.^12^ Australia’s recent SDM policy changes lie somewhere between those of the United Kingdom, Canada, and United States. SDM is now promoted through federal policy^15^ and, in some instances, also promoted at the state level. Researchers and health services have developed multiple decision aids and decision prompts.^16–23^ Healthdirect Australia, a not-for-profit, government-owned business, hosts a question prompt list “Question Builder” tool designed by the US Agency for Healthcare Research and Quality. Although the “AskShareKnow”^24^ and “Three-Talk Model”^2^ have been culturally adapted with and for Aboriginal and Torres Strait Islander populations,^25^ there are no ongoing funding streams promoting their development.^11^ Furthermore, the use of decision aids alone does not necessarily constitute SDM; there needs to be evidence-based design of implementation strategies to promote their use and sustainment.^5,26–28^ Reflections of SDM policy and implementation in these countries have highlighted the need for an overarching national policy framework that includes clear objectives, adequate and dedicated funding, and a centralized agency responsible for coordinating efforts.^12,13^

In Australia, there have been recent changes to the policy landscape promoting SDM (Table 1). The Australian Charter of Health Care Rights specifies that patients can expect to partner with health care professionals by being given the opportunity to ask questions, having access to open and honest communication, receiving clear information about risks and benefits of treatment options, and being involved in decision making.^29^ The Australian Commission on Safety and Quality in Health Care (the Commission) developed the National Safety and Quality Health Service Standards in partnership with key stakeholders.^15^ All health services in Australia are required to meet the standards to be accredited providers of health care. Recent (2019) changes to the standards require tertiary (hospital based) health services to meet specific conditions to demonstrate they have met SDM implementation criteria.^15^ Examples of evidence include education and training for clinicians to use SDM, monitoring and feedback, and documentation of SDM outcomes.^15^ An additional policy lever to promote SDM implementation in the Australian state of Victoria is the Partnering in Healthcare Framework.^30^ The Partnering in Healthcare Framework includes the following 5 domains: shared decision making, working together, personalized and holistic, effective communication, and equity and inclusion.^31^ Tertiary health services are required to focus on up to 2 Partnering in Healthcare Framework domains each year. This means that all health services across Victoria have the opportunity to work on SDM implementation at some point over 5 y (from the Framework’s inception in 2019).

**Table 1:** Policy Implementation and Practice Timeline for SDM in Australia and Study Timelines^a^

November 2017	The Australian Commission on Safety and Quality in Health Care release the second edition of the National Safety and Quality Health Service Standards
January 2019	Health service assessment to the new National Safety and Quality Health Service Standards begins
February 2019	Safer Care Victoria launches the Partnering in Healthcare Framework
April 2019	Safer Care Victoria inaugural Partnering in Healthcare Framework forum
June 2019	Health services complete statement of intent outlining which domain they will focus on for 2019–2020 The Women’s select Shared Decision Making as 1 of 2 domains of focus from the Partnering in Healthcare Framework
August 2019	The Australian Commission on Safety and Quality in Health Care launched “My Healthcare Rights,” the second edition of the Australian Charter of Healthcare Rights
November 2020	The Women’s partners with the research team to explore SDM implementation in maternity care
June 2021	Qualitative interviews with the Women’s health service staff and policy makers
February 2022	The Women’s completes health service accreditation
December 2022	The Women’s includes Shared Decision Making in their strategic plan with a vision to embed SDM across care settings

a This study took place during the COVID-19 pandemic in Melbourne, Australia. Health service implementation of Partnering in Healthcare Domains, health service accreditation, and study data collection were delayed to varying amounts.

It is unclear how hospital-based health services are reacting to and implementing these new SDM policies in practice. The last decade has seen increased growth in policies promoting SDM implementation.^13^ However, some researchers have highlighted concerns that policies promoting SDM implementation may be outpacing practice and risking unintended consequences.^32^ For example, the lack of clear definition for SDM^33^ and differences in measurement instruments^34^ means there is a risk that health services are implementing SDM to different standards.

In hospital contexts, the risk of these unintended consequences may be amplified. There is a dearth of context-specific available research for barriers and facilitators to SDM in hospital settings.^35^ Furthermore, research in hospital-based SDM tends to focus on the perceptions of clinicians and patients, without considering the perspectives of nonclinical staff.^35,36^ These staff are likely to have better oversight of organizational- and system-level factors influencing SDM implementation.^36^ As such, Australian hospitals are left with the difficult task of changing practice based on new policies with very little contextually relevant evidence to say what does work to implement SDM in routine care.

The aim of this study was to explore the barriers and facilitators to SDM implementation from the perspective of multiple stakeholders within 1 health service in Victoria, Australia, following recent changes to state and federal policies. Prior to the study, the Royal Women’s Hospital had used some implementation strategies to progress the uptake of SDM in the service, such as engaging with Safer Care Victoria and selecting SDM as 1 of 2 domains of focus from the Partnering in Healthcare Framework; staff attending Safer Care Victoria’s SDM Community of Practice^37^ and inviting the researchers to provide an education session on SDM research and implementation to their Consumer Advocacy Committee. This study was part of a larger study exploring the barriers and facilitators to SDM in the large tertiary maternity health service to co-design a behavioral intervention (including implantation strategies) to promote SDM.^38^ Importantly, this study includes stakeholders who are either responsible for implementing these new policies or whose care provision may be affected, many of whom are not usually included in hospital based SDM research.^35^

## Materials and Methods

This qualitative study used semi-structured interviews to elicit participants’ barriers and facilitators to the implementation of SDM, mapped to the Theoretical Domains Framework.^22^ We define SDM implementation as activities used by the health service to embed SDM in routine maternity care practice.

Ethics approval was obtained from The Royal Women’s Hospital, also denoted as “the Women’s,” Human Research Ethics Committee (reference HREC/73706/RWH-21-14) prior to data collection. All participants provided written informed consent. Design and reporting were based on the Theoretical Domains Framework (TDF) and COnsolidated criteria for REporting Qualitative research (COREQ)^39^ (see Supplementary Material).

### Participants

Participants included clinicians, health service administrators, health service decision makers, and government policy makers. Clinicians included midwives, allied health, and obstetricians, as well as general practitioners of varying experience. Health service administrators were staff members at the Women’s responsible for implementing and managing patient experience programs and quality and safety programs. Health service decision makers were staff responsible for leading and managing maternity services. Government policy makers were Victorian Department of Health staff with experience in SDM implementation.

Participants were recruited using purposive and snowball sampling and were invited to take part in the study via e-mail. Participants were asked to recommend the names of up to 3 others in similar roles who may have different experience to their own. Sampling aimed to obtain a broad cross section of experience and seniority levels (see Supplementary Material).

### Study Setting

#### Data collection

Interview guides were developed and tested prior to conducting interviews. Interviews were semi-structured in nature and broadly followed the TDF.^40^ Interviews were conducted between June and August 2021 online or over the phone and were 20 to 50 min in length. A.W. conducted all interviews and took field notes during interviews and while listening to recordings. Interviews were audio recorded and transcribed using an encrypted Web service.

#### Data analysis

This research study was underpinned by methods for using the TDF described by Atkins et al.^40^ The TDF was produced by behavioral scientists and groups theories of behavior into domains.^40^ It has been used widely to explore and organize the factors (barriers and facilitators) that influence behavior and behavioral intervention implementation.^40–42^ Findings from TDF can be used in conjunction with other behavioral science approaches to design interventions for changing behavior.^43^ This method has been selected for this study as it aids exploration of multiple stakeholders’ experiences to understand the motivation, environment, attitudes, and beliefs underpinning their behavior. Specifically, the TDF underpinned the interview questions, the collection and analysis of data, and reporting of themes. Importantly, the use of the Atkins et al.^40^ approach promotes both inductive and deductive coding to analyze interviews, and responses were not restricted to the TDF or specific behaviors.^44^ Inductive coding involves thematically analyzing themes that can be mapped to the TDF while also including themes that do not map to the TDF, whereas deductive coding involves using the TDF domains to guide content analysis. Rigid use of the TDF may mean important non-TDF findings are overlooked by researchers^44^; as such, this study used both inductive and deductive coding.

Data analysis was based on McLellan et al.^45^ Each transcript was read several times with audio to ensure familiarity. Two authors coded 6 transcripts using the predetermined coding guide (the TDF). Any text that was not coded was identified and coded using inductive analysis. Where conflicts occurred during coding, there was discussion until consensus was reached. Additional codes were created from inductive coding by one author and checked by the second author. The remaining transcripts were coded by one author and double-checked by the second author. Findings were discussed and interpretations challenged until consensus was reached during meetings with the author team.

## Results

Twenty-four participants were interviewed, including 13 clinicians, 6 health service administrators and decision makers, and 5 government policy makers (Table 2). Clinicians included 5 midwives, 5 doctors, and 3 allied health professionals. Midwives were a mix of team midwifery, specialist programs, and caseload. Doctors included junior and senior obstetricians and 1 general practitioner. Allied health professionals included psychology, social work, and dietetics. Three health service administrators and 3 health service decision makers participated. Five government policy makers were interviewed. TDF domains identified included Knowledge, Environmental context and resources, Reinforcement, and Memory, attention and decision-making processes, and Beliefs about consequences (Table 3).

**Table 2:** Participant Characteristics

**Clinicians**	**13**
**Midwives**	**5**
Team midwifery	2
Specialist programs (i.e., drug and alcohol services, young women’s program, Indigenous program)	2
Case load	1
**Doctors**	5^a^
Obstetrician, junior	2
Obstetrician, senior	2^a^
General practitioner	1
**Allied health**	3
Social work	1
Dietetics	1
Psychology	1
**Health service**	6^a^
Health service administrators	3^a^
Health service leaders/decision makers	3^a^
**Government policy makers**	5

a Some health service staff members had multiple roles across clinical, administration, and management. They were asked to respond from the perspective of their predominant role and appear only once in the table (i.e., senior clinicians who also hold managerial positions are counted only under the doctor cohort).

**Table 3:** Themes and Subthemes Pertaining to the Implementation of SDM in Hospital-Based Maternity Care Mapped to the Theoretical Domains Framework

Theoretical Domains Framework Domain and Subtheme	Barriers	Facilitators
Knowledge		
Awareness and understanding of SDM	• Staff and clinician limited/mixed awareness and/or knowledge of SDM • Unclear definitions of SDM in accreditation, policies, and guidelines	• Using other terminology to describe SDM when there is limited awareness/knowledge • Using a shared definition of SDM
Knowledge of SDM policy	• Clinician’s limited knowledge of SDM policy and accreditation requirements • Safer Care Victoria’s lack of resources to track how policy is being implemented	• Government policy makers’, health service administrators’, and decision makers’ in-depth knowledge of SDM policy and accreditation requirements
Environmental context and resources		
Systems approach	• Siloed or “one-off” projects to implement SDM	• Using “whole-of-system” approach to implementation • Health service’s mission statement endorses SDM
Documentation	• Lack of or poor documentation leading negatively affecting continuity of care	• Good documentation from previous clinicians enables SDM conversations • Electronic medical tecords system seen to support documentation and implementation of SDM
Reinforcement		
Measurement	• Health service need to know what accreditation surveyors are looking for to prove that SDM has been implemented (knowing measurement requirements is a facilitator)	• Measuring and reporting SDM implementation using patient outcomes facilitates further implementation
Memory, attention, and decision-making processes		
Capacity for implementation	• Health service leaders lack of time for forward planning and competing priorities, meaning SDM implementation may not be a high priority • Clinicians may not have implementation experience • Clinicians being asked to implement multiple projects at the same time	• Providing clinicians with project management staff to facilitate SDM implementation
Beliefs about consequences		
Beliefs about consequences of SDM implementation	• Policy makers’ belief that health services lack sufficient capability and capacity to implement SDM due to lack of resources and system capability • Health service staff’s belief that clinicians would not want to implement SDM as it does not align with hospital guidelines and policy • Belief that implementing SDM will mean disappointing patients when the service cannot meet their needs	

SDM, shared decision making.

### Knowledge

#### Awareness and understanding of how SDM influences implementation

Participants felt a major barrier to the implementation of SDM was the limited awareness and mixed definitions of SDM held by colleagues. Some participants reported colleagues having no awareness of the term *shared decision making*.

I went in there and I cold called it, “Tell me about your shared decision-making projects. Tell me where it’s happening,” we kind of get crickets [silence]. (Policy maker 5)

I don’t know that it’s a term or concept, or at least the term for that concept that is commonly used. (Health service decision maker 2)

Some participants felt implementation efforts were hampered by unclear definitions used in accreditation standards, in policy and guideline documentation and clinicians’ belief they were doing it already.

I noticed in the standards, even though the standard talks about partner with consumers and they talk about shared decision making, when they describe the evidence, they don’t use that terminology. (Policy maker 5)

The thing that got in their [clinicians’] way was their realm of awareness and their realm of understanding in relation to what their current practice was and thinking that they were doing shared decision making. (Policy maker 4)

To overcome these barriers, some policy makers and health service staff reported using other terms to speak about SDM. They felt it was important to describe SDM or use other terminology as SDM was not well known or understood.

We started the conversation about shared decision making and called it *partnership*. That just seems to be the language that resonates here. (Health service decision maker 2)

I wonder if the terminology might be ahead of the actual practice in health services. (Policy maker 5)

Others felt it would be important to develop and use a shared definition of SDM to raise awareness and ensure consistent implementation efforts.

I think you do need to have guidelines and governance around that. What is the hospital’s stance on this topic? . . . If you’re doing orientation programs for junior staff, you could include [the definition] in the orientation pack. You could do education on how to have a conversation. (Obstetrician 1)

Have role play or modeling exercises where you can try to demonstrate exactly the principles of it are very helpful. (Health service decision maker 3)

#### Awareness of SDM policy

Awareness of SDM implementation policies was mixed among participants. Government policy makers had an in-depth awareness of policies. Likewise, health service staff knew the Partnering in Healthcare Framework and accreditation requirements. In contrast, clinicians had little knowledge of SDM policy and often had no knowledge of Safer Care Victoria. Safer Care Victoria is the Victorian state agency responsible for monitoring quality and safety in health services and developed and monitors the Partnering in Healthcare Framework.

There’s probably not enough visibility of Safer Care and what they believe we should be doing amongst clinicians. (Obstetrician 1)

I would say majority of my exposure has been through the launch of the Partnering in Healthcare framework from Safer Care Victoria. And I guess every health service needs to commit to a priority in one or two of those. (Health service administrator 1)

Safer Care Victoria had no way of knowing how their Partnering in Healthcare Framework was being implemented by health services.

Nothing that we do is enforceable or mandated in any way. I can provide evidence-based guidelines that goes on our Internet, but it’s largely down to the hospitals to whether they choose to implement that guidance. (Policy maker 1)

### Environment Context and Resources

#### Systems approach

Health service administrators and decision makers and policy makers spoke about the importance of using a systems-based or whole-of-service–based approach to SDM. They felt it was crucial to implement SDM across the service, rather than through siloed projects. They felt their strategy and mission statements helped, as they let staff and patients know it was a priority for the whole organization.

The risk of having a specific project just about SDM, unless it’s going to be a whole of organization philosophy in terms of how we practice, there’s no point. (Policy maker 4)

. . . shouldn’t be just maternity focused. It should be something, this is what we do at the Women’s . . . it is part of the patient experience. (Health service decision maker 1)

This view was reinforced by many of the clinicians who said they felt supported by the health service to practice SDM because it was in line with the mission statement and strategic plan of the Women’s.

Within the strategic plan and then within the way the hospital values are, there is an emphasis on family centred, patient centred care. And I think that SDM comes as part of that. (Midwife 3)

#### Documentation

Clinicians spoke about the importance of good documentation (i.e., accurately documenting which options were discussed and women’s preferences and goals). Lack of documentation or poor documentation was seen as a barrier to implementing SDM for clinicians, health service administrators, and decision makers. Health service leaders saw a lack of documentation as a barrier for subsequent clinicians building relationships and ensuring a continuity of care between appointments. Clinicians supported this view; it was crucial to access information from other clinicians to prepare for SDM discussion with patients.

But I think that lots of patients feel like they have to come here and repeat themselves, time and time again, around what their preferred options are. Because either as staff aren’t reading that information or they’re not documenting it correctly, or they just don’t care. (Health service administrator 1)

Health service decision makers and clinicians were optimistic about the potential of electronic medical records (EMR) system in supporting documentation and the implementation of SDM.

Documentation is a lot easier now with an electronic medical record system. (Obstetrician 4)

We really [need to] try to make the EMR work for us. I don’t know if functionalities within electronic medical records have ever been looked at as ways of promoting shared decision making. (Health service decision maker 3)

### Reinforcement

#### Measurement

Health service administrators and decision makers felt it was crucial to measure and report how SDM is being implemented. Measurement could be conducted through different mechanisms, but specifically, reporting patient outcomes was seen as key outcome that would facilitate SDM.

I think the enablers or the things that sort of help it happen is if the concept is promoted and supported in a way that is feasible and that the value of it is highlighted in terms of patient outcomes. (Health service decision maker 3)

While policy makers felt it was crucial to measure and report on how SDM is being implemented. One policy maker noted the importance of health service staff understanding how to provide evidence of SDM for the National Safety and Quality Health Service Standards accreditation, as surveyors would be looking for specific examples of how SDM is part of their practice.

But the surveyors during accreditation . . . want to see evidence in the health records, whether it’s electronic health records, they want to see records of conversations, they want to see audits of those records and indicators that we’ve set reasonable targets for SDM. (Policy maker 5)

### Memory Attention and Decision-Making Process

#### Capacity for implementation

Health service administrators and decision makers felt that health service leaders’ lack of time for forward SDM implementation planning was a barrier to SDM. They felt that the leaders were too busy and many had competing priorities that meant SDM was not a high priority.

I don’t see a lot of really strong clear leadership from the leadership team in terms of advocating for change, talking about change. Talking about the way, the reasons why we need to change and supporting staff to do that. I think our leaders in maternity, whilst they’re doing the absolute best job they can, they are very operational and they are very in the moment here and now and sometimes resistant to change. And I think that stems a lot from the demand that that group is under. (Health service administrator 1)

They also felt that clinicians did not necessarily have the time or experience with project implementation and that they needed to be supported by a project manager who would be able to guide the project through all phases.

[Clinician’s] expertise, is doing the work. But then to say, go and do a project, or implement that pathway, they can do it, but they do need guidance, and probably a project person . . . I think that project support is really important until you get that implementation. (Health service administrator 2)

Policy makers were acutely aware of clinicians being asked to implement multiple projects at a time. They felt this was a barrier for all project implementation, including SDM, as staff were overburdened and project effectiveness was quickly diluted with additional projects.

It’s an overcrowded clinical space. And yet, here’s another intervention, and here is another thing that you should be doing. (Policy maker 2)

### Beliefs about Consequences of SDM Implementation

Both health service staff and policy makers feared negative outcomes of SDM implementation efforts. Policy makers were concerned that health services would not have the capability and capacity to make the necessary changes to implement SDM. They also believed health services would push back on SDM implementation due to a lack of resources and system capability. Similarly, health service staff believed clinicians would push back against SDM implementation as it did not align with hospital guidelines and policy. There were also concerns that implementing SDM would risk changing women’s expectations of care, which the system might not be able to support, especially when it deviated from standard care.

There’s that [health service] barrier of, “We have to reschedule, reprogram, reimagine this delicate balance of 15 layers of computer programmes that we’ve got.” (Policy maker 3)

I think that it would be a big kind of cultural change, and I think you run, I suppose, the risk of more women not wanting to have their care delivered in a way that aligns with our guidelines. (Health service administrator 3)

I guess don’t go too far and don’t set expectations with patients that aren’t necessarily there yet. (Health service administrator 1)

## Discussion

This study was conducted following the release of policies requiring SDM implementation in tertiary health services in Australia. To our knowledge, this is the first Australian study of organizational- and system-level barriers and facilitators to SDM implementation in tertiary maternity care to include perspectives beyond patients and clinicians.^46,47^ Five TDF domains emerged: knowledge, environment context and resources, reinforcement, memory attention and decision-making processes, and beliefs about consequences. The barriers and facilitators elicited subsequently informed intervention development at the Women’s.^38^

Health service administrators and decision makers reported using different ways to describe SDM without using the term to overcome the lack of or mixed understanding. This method allowed them to create a shared understanding of what SDM involved in practice. However, some insisted it was crucial to develop a shared definition to facilitate SDM implementation. This supports the concerns of researchers who warn that a lack of a clear, shared definition of SDM will impede the implementation of SDM policy in practice.^32^

Nonclinical staff are rarely included in SDM and SDM maternity care research.^35,36,46,48^ These results further support the idea that nonclinical staff have important insights into the barriers and facilitators affecting SDM implementation and should be included.^38,49^ For example, policy makers, health service administrators, and decision makers were aware of the relevant SDM policy and had insight into the organizational- and system-level factors that clinicians did not. There was a concentrated awareness at the policy level and less awareness at the clinician level, with health service administrators and decision makers in the middle. As such, there is a clear knowledge translation role for health service administrators and decision makers to help close the policy to practice gap.^50^

Environmental context and resources were reported as a crucial factor for SDM implementation. This is an often-reported factor in the research literature.^35,36,51,52^ A novel finding of this research is that participants indicated that a systems approach to SDM, rather than siloed or once-off projects, should be the focus of implementation. A systems approach could include, for example, changes to orientation packs for junior staff to include SDM, embedding SDM into EMRs, and an updated strategic direction, indicating the whole service is working on implementing SDM. Whereas an example of a siloed approach would be using a decision aid for one decision point in a clinical area, a commonly researched area Australian maternity care.^18–20^ Policies mandating SDM implementation support a systemwide approach in Australian tertiary health services.^15^ However, research to date has not directly assessed the outcomes of such policies. Policy makers, health service administrators, and decision makers were acutely aware of health service capacity for implementation. All felt it was crucial to the success of SDM implementation to provide specific staff to manage projects rather than relying on clinicians who do not have capacity.

Health service staff and clinicians viewed the health service’s mission statement and strategic plan as drivers of the whole of service approach. Documentation was seen as a key facilitator of SDM. Handheld paper-based records were seen to facilitate SDM in maternity services in Australia.^53^ In this study, respondents felt that the use of EMRs could better support clear communication between clinicians and patients. Previous studies suggest that not embedding SDM in EMRs workflows may inhibit implementation.^54^ EMRs are new additions to public health services in Australia.^55^ The arrival of EMRs in health services provides an exciting resource for SDM implementation at a service-wide level through documentation and workflows.

Health service administrators, decision makers, and policy makers all called for better measurement of SDM, including its influence on patient outcomes. Research highlights key issues with current SDM measurement instruments.^34,56^ Measurement instruments lack evidence for their measurement quality due to poor reporting or lack thereof.^34^ The perspective (patient, clinician, observer) taken during measurement affects the association found between SDM and patient outcomes.^56,57^ There is low agreement across measures taken from different perspectives.^58^ This may be due in part to different understandings of what SDM involves.^58^ The characteristics of instruments also vary greatly (e.g., population of interest, constructs used, application, items scored, and perspective), and there are more than 40 SDM instruments.^34,56^ Ceiling and halo effects are also an issue in SDM measurement. Some measures find SDM scores at their maximum (ceiling effect),^34^ whereas others may entangle other experiences of care (halo effect).^59^ This leaves health services with a wide variety of measures to select from, leading to a risk of inappropriate selection, analysis, and the potentially incorrect perception that SDM has already been implemented to a high standard.

At the system level, the Commission does not report how health services are meeting each action item under the accreditation Standards, instead reporting aggregate data.^11,60^ In Victoria, SDM is measured by the Victorian Health Experience Survey, which includes 5 questions on SDM.^61^ These survey questions do not constitute a validated measure of SDM and therefore does not measure SDM appropriately (although it is an improvement on the previous version, which included fewer questions on SDM^62^). As such, policy makers report they have no way of explicitly measuring whether health services respond to SDM implementation policy.

This study reports important nuances in the policy to practice gap implementing SDM in hospital-based maternity care. A major strength of this study is the inclusion of nonclinical staff to understand the organizational- and system-level barriers and facilitators to SDM. Study rigor was established with the use of multiple perspectives, inductive and deductive analysis of codes and themes, and through ongoing peer checking and iteration.^63^ SDM research is often criticized for its lack of theory and unclear reporting.^64^ A strength of this study is the use of the TDF and COREQ for the systematic design, data collection, analysis, and reporting. The use of the TDF allows for future intervention research to build on the available behavioral science evidence base.

However, there are some important limitations. Our sample may have been biased toward health service administrators, decision makers, and policy makers who have a greater understanding of SDM. This study also did not include the perspectives of women or their families. Further research should aim to include women and their families and staff members with limited understanding or unfavorable opinions of SDM. Furthermore, this study was conducted at one metropolitan health service. Future research should aim to include multiple sites with different contexts (e.g., regional, remote, and private health services).

## Conclusion

This study is the first to our knowledge to explore barriers and facilitators to SDM implementation following the release of Australian policies mandating SDM implementation. To understand organizational and system factors, the study gathered the perspectives of clinicians and perspectives not usually included in SDM research: health service decision makers, administrators, and government policy makers. Awareness of SDM policies varied, with clinicians having the least awareness compared with other participants. Participants ultimately wanted to see a universal definition used and promoted. All participants felt SDM should be implemented as whole-of-service approach, rather than once-off projects. This is an important and novel finding. Participants felt this could be supported through the integration of SDM across EMRs to enable SDM documentation and workflows. Measurement was an important facilitator for SDM implementation, especially the use of patient outcome measures.

Further research is needed to understand how health services are implementing SDM in practice at the national level. Notably, there are a multitude of health services currently attempting to implement SDM in practice to meet policy requirements. However, it is unclear how SDM is being measured during accreditation and therefore to what extend SDM is being implemented. Without this oversight and reporting mechanisms, important learnings are likely to be lost. The Commission is well-placed to collect, analyze, and disseminate learnings from SDM implementation in hospitals in line with their policy mandates. More broadly, this research adds to calls for implementation research to understand what does work to implement SDM in hospitals, to use agreed-upon definitions and measures of SDM, and to involve multiple stakeholders. Finally, nonclinical staff seem to play a crucial role in translating policy to practice and should be included in SDM implementation research.

## Supplemental Material

sj-docx-1-mpp-10.1177_23814683231199943 – Supplemental material for “The Terminology Might Be Ahead of Practice”: Embedding Shared Decision Making in Practice—Barriers and Facilitators to Implementation of SDM in the Context of Maternity Care Click here for additional data file. Supplemental material, sj-docx-1-mpp-10.1177_23814683231199943 for “The Terminology Might Be Ahead of Practice”: Embedding Shared Decision Making in Practice—Barriers and Facilitators to Implementation of SDM in the Context of Maternity Care by Alex Waddell, Denise Goodwin, Gerri Spassova and Peter Bragge in MDM Policy & Practice
