# Supplementary material for: “The Terminology Might Be Ahead of Practice”: Embedding Shared Decision Making in Practice—Barriers and Facilitators to Implementation of SDM in the Context of Maternity Care
Source: MDM Policy Pract. 2023 Sep 22;8(2):23814683231199943. doi: 10.1177/23814683231199943 (PMC10517621; doi:10.1177/23814683231199943)
Supplement: sj-docx-1-mpp-10.1177_23814683231199943 – Supplemental material for “The Terminology Might Be Ahead of Practice”: Embedding Shared Decision Making in Practice—Barriers and Facilitators to Implementation of SDM in the Context of Maternity Care [file sj-docx-1-mpp-10.1177_23814683231199943.docx]

**Supplementary Material**

1. Recruitment
2. Qualitative Analysis
3. Table 2: COREQ checklist
4. **Recruitment**

Health Service Staff

Purposive and snowball sampling techniques were used to select health service staff, including clinicians, health service administrators, and decision makers (2). In line with the aim of the study, the purposive sampling approach included respondents with different lived experience of SDM implementation including health service staff involved in patient care, decision making, and/or administration. We based our anticipated sample size on a sample of n=5 to n=20 per cohort (1, 2). During the interviews, participants were requested to suggest up to three individuals in their cohort who held different seniority levels and/or practiced in different areas to ensure we were including different perspectives. Data saturation was operationalised as the point at which each interviews with each cohort were not producing new barriers and facilitators (1). In this study data saturation was reached with the anticipated sample sizes for each cohort.

Some health staff members hold multiple roles across clinical, administration, and management. For example, a senior obstetrician may also hold a managerial role within the health service. These staff members were asked to respond from their predominant role (i.e. from their role as an obstetrician rather than their role as a manager).

Policy Makers

Purposive and snowball sampling techniques were used to select policymakers. The first government policymakers was known to the researchers and was invited to participate due to their extensive experience in policy and framework development, including SDM implementation policy development. Subsequent policymakers were identified through snowballing, with participants asked to provide up to two names of individuals with expertise in SDM implementation in health service settings, or SDM in maternity policy and guideline development. These individuals were then invited to participate in the study. Data saturation was operationalised using the same approach as health service staff and the anticipated sample size was reached.

1. **Qualitative Analysis**

The following steps were taken to analyse the data based on McLellan et al. (2019) (3):

1. Transcripts were uploaded to qualitative analysis tool NVivo.
2. AW read each transcript with audio several times to ensure familiarity.
3. Transcripts were coded using the direct content analysis approach (39). Six transcripts (one from each cohort) were coded by DG, using the predetermined coding guide. Any text that could not be coded was identified to be coded and analysed in step 4 (39). Where conflicts occurred in coding, there was discussion until consensus was reached.
4. Additional codes were created from the un-coded text in step 3 by AW, codes were checked by DG.
5. The remaining transcripts were coded by AW with assistance from DG who double checked any codes AW was unsure of.
6. Findings were discussed and interpretations challenged until consensus was reached during meetings with the author team.
7. **Table 2: COREQ checklist**

**The Consolidated Criteria for Reporting Qualitative Studies (COREQ): 32-item checklist**

Developed from: Tong A, Sainsbury P, Craig J. Consolidated criteria for reporting qualitative research (COREQ): a 32-item checklist for interviews and focus groups. International Journal for Quality in Health Care. 2007. Volume 19, Number 6: pp. 349 – 357

| **No. Item** | **Guide questions/description** | **Page reported on**  **Notes** |
| --- | --- | --- |
| **Domain 1: Research team and reﬂexivity** | | |
| ***Personal Characteristics*** | |  |
| 1. Inter viewer/facilitator | Which author/s conducted the interview or focus group? | AW |
| 2. Credentials | What were the researcher’s credentials? E.g. PhD, MD | BSc, MPH |
| 3. Occupation | What was their occupation at the time of the study? | PhD Candidate |
| 4. Gender | Was the researcher male or female? | Female |
| 5. Experience and training | What experience or training did the researcher(s) have? | The interviewing researcher has a public health and psychology background with experience in qualitative research. |
| ***Relationship with participants*** | | |
| 6. Relationship established | Was a relationship established prior to study commencement? | The researcher had a working relationship with the first government policy maker interviewed. |
| 7. Participant knowledge of the interviewer | What did the participants know about the researcher? e.g. personal goals, reasons for doing the research | Participants were informed that the researcher was interested in exploring the barriers and facilitators to SDM as part of her PhD. |
| 8. Interviewer characteristics | What characteristics were reported about the inter viewer/facilitator? e.g. Bias, assumptions, reasons and interests in the research topic | Participants were informed that the researcher was interested in exploring the barriers and facilitators to SDM and how these might align across cohorts to as part of her PhD. |
| **Domain 2: Study design** | | |
| ***Theoretical framework*** | | |
| 9. Methodological orientation and Theory | What methodological orientation was stated to underpin the study? e.g. grounded theory, discourse analysis, ethnography, phenomenology, content analysis | 4  In line with the phenomenological approach, the researchers used bracketing at the beginning of the research project to discuss and put aside their own ideas and biases of the research question (1). |
| ***Participant selection*** | | |
| 10. Sampling | How were participants selected? e.g. purposive, convenience, consecutive, snowball | 4 |
| 11. Method of approach | How were participants approached? e.g. face-to-face, telephone, mail, email | 4 |
| 12. Sample size | How many participants were in the study? | 4 |
| 13. Non-participation | How many people refused to participate or dropped out? Reasons? | All health service staff (n=9) who were invited but did not participate did so due to lack of time with clinical or other demands. |
| ***Setting*** |  |  |
| 14. Setting of data collection | Where was the data collected? e.g. home, clinic, workplace | 4 |
| 15. Presence of non-participants | Was anyone else present besides the participants and researchers? | Page 4 |
| 16. Description of sample | What are the important characteristics of the sample? e.g. demographic data, date | Supplementary material |
| ***Data collection*** |  |  |
| 17. Interview guide | Were questions, prompts, guides provided by the authors? Was it pilot tested? | Page 4 |
| 18. Repeat interviews | Were repeat inter views carried out? If yes, how many? | No repeat interviews were conducted. |
| 19. Audio/visual recording | Did the research use audio or visual recording to collect the data? | Page 4 |
| 20. Field notes | Were ﬁeld notes made during and/or after the interview or focus group? | The researcher took field notes during the interviews. |
| 21. Duration | What was the duration of the interviews or focus group? | Page 4 |
| 22. Data saturation | Was data saturation discussed? | Page 4 |
| 23. Transcripts returned | Were transcripts returned to participants for comment and/or correction? | All participants were provided with the option to read and comment / amend the transcript of their interview. |
| **Domain 3: Analysis and findings** | | |
| ***Data analysis*** | | |
| 24. Number of data coders | How many data coders coded the data? | One researcher coded the entire dataset, with a subset (18%) double coded by a second researcher. |
| 25. Description of the coding tree | Did authors provide a description of the coding tree? | Themes and subthemes are clearly labelled throughout the manuscript. |
| 26. Derivation of themes | Were themes identified in advance or derived from the data? | Page 5 |
| 27. Software | What software, if applicable, was used to manage the data? | NVivo was used to manage the data. |
| 28. Participant checking | Did participants provide feedback on the findings? | Participants did not provide feedback on the findings. |
| ***Reporting*** | | |
| 29. Quotations presented | Were participant quotations presented to illustrate the themes/ findings? Was each quotation identified? E.g. participant number | Results section |
| 30. Data and findings consistent | Was there consistency between the data presented and the findings? | Quotes presented are consistent between the results and the discussion. |
| 31. Clarity of major themes | Were major themes clearly presented in the findings? | Results section. |
| 32. Clarity of minor themes | Is there a description of diverse cases or discussion of minor themes? | Yes, cohort specific minor themes and diverse cases are discussed throughout the results and discussion. |

References:

1. Creswell JW, Poth CN. Qualitative Inquiry and Research Design: Choosing Among Five Approaches. 2017.

2. Polkinghorne DE. Phenomenological Research Methods. 1989.

3. McLellan JM, O’Carroll RE, Cheyne H, Dombrowski SU. Investigating midwives’ barriers and facilitators to multiple health promotion practice behaviours: a qualitative study using the theoretical domains framework. Implementation Science 2019 14:1. 2019;14(1):1-10.
